# Mobile Heart Rate Variability Biofeedback Improves Autonomic Activation and Subjective Sleep Quality of Healthy Adults – A Pilot Study

**DOI:** 10.3389/fphys.2022.821741

**Published:** 2022-02-17

**Authors:** Benedict Herhaus, Adrian Kalin, Haralampos Gouveris, Katja Petrowski

**Affiliations:** ^1^Medical Psychology and Medical Sociology, University Medical Center of the Johannes Gutenberg University Mainz, Mainz, Germany; ^2^Sleep Medicine Center and Department of Otolaryngology, University Medical Center of the Johannes Gutenberg University Mainz, Mainz, Germany; ^3^Department of Internal Medicine III, University Hospital Carl Gustav Carus Dresden, Technical University Dresden, Dresden, Germany

**Keywords:** heart rate variability (HRV), biofeedback (BF), 0.1 Hz breathing, sleep, actigraphy

## Abstract

**Objective:**

Restorative sleep is associated with increased autonomous parasympathetic nervous system activity that might be improved by heart rate variability-biofeedback (HRV-BF) training. Hence the aim of this study was to investigate the effect of a four-week mobile HRV-BF intervention on the sleep quality and HRV of healthy adults.

**Methods:**

In a prospective study, 26 healthy participants (11 females; mean age: 26.04 ± 4.52 years; mean body mass index: 23.76 ± 3.91 kg/m^2^) performed mobile HRV-BF training with 0.1 Hz breathing over four weeks, while sleep quality, actigraphy and HRV were measured before and after the intervention.

**Results:**

Mobile HRV-BF training with 0.1 Hz breathing improved the subjective sleep quality in healthy adults [*t*(24) = 4.9127, *p* ≤ 0.001, *d* = 0.99] as measured by the Pittsburgh Sleep Quality Index. In addition, mobile HRV-BF training with 0.1 Hz breathing was associated with an increase in the time and frequency domain parameters SDNN, Total Power and LF after four weeks of intervention. No effect was found on actigraphy metrics.

**Conclusions:**

Mobile HRV-BF intervention with 0.1 Hz breathing increased the reported subjective sleep quality and may enhance the vagal activity in healthy young adults. HRV-BF training emerges as a promising tool for improving sleep quality and sleep-related symptom severity by means of normalizing an impaired autonomic imbalance during sleep.

## Introduction

Around eighty percent of the German working population report having problems falling asleep and/or sleeping through the night (Marschall et al., 2020). For the period from 2010 to 2020, the diagnosis of insomnia rose by sixty percent in the German working population (Marschall et al., 2020). Sleep disorders are related to increased stress reactions, somatic pain, reduced quality of life, emotional stress and mood disorders as well as cognitive, memory and performance deficits (Medic et al., 2017). Besides short term consequences, having sleep disorders over the course of one’s lifetime is associated with the development of arterial hypertension, cardiovascular disease, metabolic syndrome, and type 2 diabetes mellitus (Li et al., 2021; Lu et al., 2021; Schipper et al., 2021; Zhang et al., 2021). An ever increasing number of adults are using hypnotics or sedatives to alleviate their disrupted sleep. In a US survey, 4% of the adults aged 20 and over reported using prescription sleep medication during one month (Chong et al., 2013). Most sleep medications have a number of side effects (Watson et al., 2012) and are related to a threefold increased risk of death (Kripke et al., 2012). In recent years, more and more non-pharmacological treatment options, such as cognitive behavioral therapy, proved to be quite effective in the treatment of sleep disorders (Lie et al., 2015; Edinger et al., 2021).

Heart rate variability-biofeedback (HRV-BF) is a form of cardiorespiratory feedback training intervention during which an individual can actively influence autonomous unconscious body processes (Lehrer and Gevirtz, 2014) and is often connected to 0.1 Hz breathing resulting from five seconds inspiration/five seconds expiration. Based on the synchronization of the 0° phase relation between heart rate and breathing (called the highest amplitude of respiratory sinus arrhythmia; RSA) as well as the 180° phase relationship between heart rate and blood pressure, gas exchange efficiency at the alveoli and baroreflex activity are improved by HRV-BF with paced breathing (Lehrer and Gevirtz, 2014). There is evidence that HRV-BF improves the HRV and symptom severity of disorders like hypertension, asthma, and depression (Lehrer et al., 2020).

Interestingly, an association between the HRV as a surrogate marker of autonomic nervous system (ANS) activity and sleep stages has been observed. In healthy individuals, there is a shift from sympathetic to parasympathetic dominance during non-rapid eye movement (non-REM) sleep with a temporary increase in sympathetic system activity during REM sleep (Trinder et al., 2001). Furthermore, the RSA as one mechanism of the HRV-BF has been described as an intrinsic resting function which increases during non-REM sleep (Bonnet and Arand, 1997). These pathways support the hypothesis of playing an important role of parasympathetic dominance for restorative sleep (Hayano and Yasuma, 2003) and of sleep-related vagal activation for promoting anti-inflammatory processes during sleep (Olshansky, 2016). Therefore, it may be argued that HRV-BF with paced breathing both during daytime and before going to sleep may have the potential to improve sleep quality by means of enhancing the baroreflex activity and maximizing the RSA.

Concerning the effect of HRV-BF intervention on sleep quality, four studies have demonstrated an improvement in the sleep quality of different sleep disorders (Lin et al., 2019; Park et al., 2019; Burch et al., 2020; Hasuo et al., 2020). However, only one of them used an objective sleep quality evaluation method, namely actigraphy (Park et al., 2019). The other three studies only assessed sleep-related reported outcomes depicting the respective subjective perceptions of study participants. Due to the fact that the mean age of the participants in three of these studies was greater than sixty years, the reported results may be more relevant in reference to older adults. Given that sleep disorders may be quite prevalent also in the younger adult population (Grobe et al., 2019), and due to the relative paucity of studies on the potential effects of HRV-BF on the sleep quality of healthy adults, further research is needed.

Therefore, in the current study, subjective sleep-related reported outcomes and objective actigraphic metrics of sleep quality as well as HRV during short-term resting conditions were collected prior to and following a four-week mobile HRV-BF intervention with 0.1 Hz breathing in healthy adults. Based on the studies by Hasuo et al. (2020) and Lin et al. (2019) who reported an improved subjective sleep quality after HRV-BF and the absence of any effect of paced breathing on polysomnography-based objective outcomes in self-reported good sleepers in the study by Tsai et al. (2015), we hypothesized that HRV-BF with 0.1 Hz breathing would accordingly increase the subjective but not the objective sleep quality in healthy adults (hypothesis 1). Based on the meta-analysis by Lehrer et al. (2020) that showed an HRV increase after HRV-BF in different populations, an HRV improvement after HRV-BF with 0.1 Hz breathing was to be expected in our study cohort as well (hypothesis 2).

## Materials and Methods

### Study Participants

The study participants were recruited through online tendering and notice boards at different universities. After recruitment, study inclusion and exclusion criteria were checked in a standardized interview using the Structured Clinical Interview both for axis I and II (Wittchen et al., 1997). Exclusion criteria were age lower than 18 years and higher than 65 years, BMI lower than 18.5 kg/m^2^ and higher than 27 kg/m^2^, any acute and/or chronic medical illness, mental disorders, medication affecting the heart rate or the central nervous system (e.g., beta blockers), illicit drug abuse, stressful life-events within the previous six months. The prospective participants took part in telephone interviews based on the entire procedure of the Structured Clinical Interview (SCID; Wittchen et al., 1997) according to the Diagnostic and Statistical Manual of Mental Disorders (DSM-IV; American Psychiatric Association [APA], 2000).

### Study Design

**FIGURE 1:**
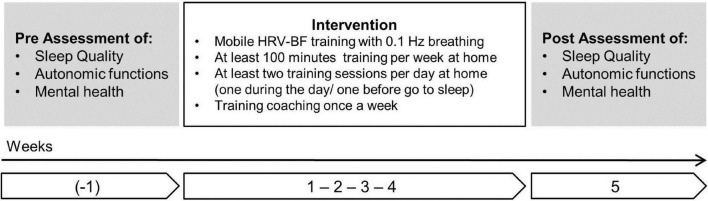
Study design of HRV-BF intervention.

The participants were instructed to perform mobile HRV-BF training with 0.1 Hz breathing at home via an ambulatory HRV-BF training system over the course of four weeks. They were further instructed to train for at least 100 min per week, including at least five daytime training sessions and five training sessions before going to sleep. At the end of each week of training, the participants were to receive a coaching telephone call to obtain feedback, technical and emotional support from a therapist. The assessment of sleep quality, autonomic activity and well-being took place before and after the four-week-training period. A detailed description of the study design is given in Figure 1. The study protocol was approved by the Ethics Committee of the Local Medical Association (Nr. 2021-16151). All study procedures were in accordance with the Principles of the Declaration of Helsinki for studies with human participants.

### Heart Rate Variability Biofeedback

The mobile system “eSense Pulse” by Mindfield Biosystems Ltd. (Gronau, Germany) was used for HRV-BF training. The participants wore a chest strap with a small sensor during training and started the training via the eSense app on their smartphone or tablet. During training, a ball that expands (breathe in) and shrinks (breathe out) on the screen specified the breathing frequency goal to a value of six cycles per minute (five seconds inspiration, five seconds expiration). The HRV was visualized by new symbols varying in color depending on the respectively measured values, which appeared every ten seconds. When the participants achieved the specified breathing frequency, resulting in an increased sinus arrhythmia, the symbols appearing on the smartphone display were green. When the participants did not achieve the specified breathing frequency, the symbols appeared in yellow (non-significant deviation), orange (significant deviation), or red (very significant deviation). The participants were instructed to adjust their breathing frequency to reach emergence of as many green symbols as possible for the entire training session. During training, the HRV was measured by a single-channel electrocardiograph (ECG) signal recorded by the chest strap at a sample rate of 500 Hz. At the end of each session, the data were either directly e-mailed from the app to the researcher or saved in the participant’s e-sense account. All data from the app were anonymized. The ECG recordings were analyzed with Kubios software (Kubios Oy, Kuopio, Finland). To eliminate extra beats in the R-R interval, an automatic filtering process method was applied (threshold: 0.45 s).

### Outcomes

#### Heart Rate Variability Measurements

For the assessment of the ANS activity before and after the training period of four weeks, two short-term measurements were carried out at the medical facility: (1) sitting in an upright position while watching the documentary ‘World below the Sea’ on the screen for five minutes, and (2) resting condition in supine position for 5 min. A single-channel electrocardiograph (ECG) signal (2 electrodes) with a sample rate of 500/s. was worn during the different measurements. The HRV system HRVScanner™ (BioSign, Ottenhofen, Germany) was used for measurement, analysis and calculation of the heart rate, heart rate variability, and pulse wave velocity. All recorded ECGs (26 participants × 4 measurements = 104) were checked by experienced personnel beat-to-beat. Artifacts were corrected using the deletion method. The rate of losses (number of heartbeats marked as artifact or ectopic beats divided by all heartbeats detected, in%) was <0.06%. HRV was quantified by the following metrics: root mean square successive differences (rMSSD), standard deviation of all NN-intervals (SDNN), total power (TF), power in the very-low frequency range 0.003–0.04 Hz (VLF), power in the low-frequency band 0.04–0.15 Hz (LF), absolute power in the high frequency range 0.15–0.4 Hz (HF) and LF/HF ratio.

#### Mental Health

Mental health – related reported outcomes were assessed using validated established questionnaires. The Short-Form 12 Healthy Survey (SF-12) measures general health and well-being as part of the SF-36 Health Survey (Morfeld et al., 2011). The two components of physical and mental health are calculated. The German version of the Perceived Stress Scale (PSS; Schneider et al., 2020) is used to measure the degree of stressful situations in one’s life during the previous month. Ten items must be answered on a 5-point scale ranging from 1 ‘never’ to 5 ‘very often’. The Brief Symptom Inventory 18 (BSI-18; Franke, 2000) captures physical and psychological complaints of the previous seven days. Based on 18 items on a 5-point scale (from ‘not at all’ to ‘extremely severe’), the dimensions of somatization, depression and anxiety are calculated.

#### Sleep Quality (Actigraphy & PSQI)

Objective sleep quality was measured using a two-night wrist actigraphy. The participants were instructed to wear the wrist actigraphy on their non-dominant hand. Additionally, the time points of going to sleep, waking up at night, and waking up in the morning were to be marked. Also, sleep diaries were to be kept. Based on actigraphic recordings, total sleep time (TST), sleep onset latency (SOL), number of awakenings (NAW), and waking after sleep onset (WASO) were analyzed. The Motionlogger ^®^ (Ambulatory Monitoring Inc., Ardsley, United States) actigraph was used and was set to record data in 10-s epochs. Action-W Version 2.7.3045 software (AW2.7, Ambulatory Monitoring Inc., Ardsley, United States) was used to analyze the sleep patterns.

Subjective sleep quality was measured by the German version of the Pittsburgh Sleep Quality Index (PSQI; Riemann and Backhaus, 1996). This questionnaire assesses sleep quality over a one-month-period using ten items. More specifically, the PSQI measures sleep quality, sleep latency, sleep duration, habitual sleep efficiency, sleep disturbances, use of sleep medication, and daytime dysfunction. Each item is answered on a four-point scale ranging from ‘not at all’ to ‘3× or more per week’.

### Statistical Analysis

Sample size was calculated with the G*power program (version: 3.1.9.2.) (Faul et al., 2007). Previous HRV-BF studies demonstrated a small to medium effect size [see meta-analysis by Lehrer et al. (2020)]. Power analysis showed that for an expected medium effect size of Cohen’s *d* = 0.5 for the HRV outcome measure using a dependent *t*-test to prove pre- and post- measurement at a significance level of *p* = 0.05 and power of 80% (1-ß = 0.80), a total sample size of *n* = 27 participants would be needed. The software program used to analyze the data was *SPSS Statistics version 23* (IBM, Chicago, IL, United States).

The effects of HRV-BF intervention on heart rate, HRV parameters, psychological measures, and sleep quality were analyzed by a dependent t-test. The HRV values were transformed by logarithm naturalis + 1 to obtain approximately normal distributions.

## Results

### Participants

The data collection from the 27 individuals included in the study was conducted between November 2020 and September 2021. One participant dropped out during the intervention period. The mean age of the 26 healthy participants (15 women and 11 men) was 26.04 ± 4.52 years, and the mean body mass index was 23.76 ± 3.91 kg/m^2^.

### Heart Rate Variability-Biofeedback Training

**FIGURE 2:**
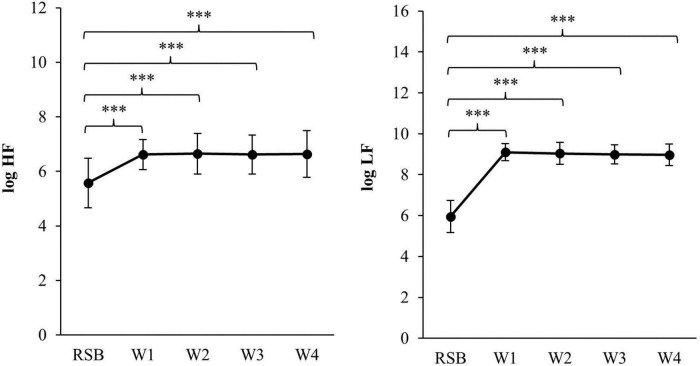
Changes in autonomic function showing in HRV-parameters log HF and log LF (M ± SD) during base line and four weeks of intervention with 0.1 Hz breathing. RSB, Resting sitting position with spontaneous breathig; W1, week 1; W2, week 2; W3, week 3; W4, week 4. ^***^p ≤ 0.001.

Over the four intervention weeks, the participants on average performed 21.04 (SD = 2.91) daytime training sessions and 20.56 (SD = 3.32) training sessions before going to sleep. The mean training time was 103.80 (SD = 9.56) minutes/week. Figure 2 demonstrates successful HRV-BF training with an increase in the HRV-parameters ln + 1 HF and ln + 1 LF over the course of four weeks.

### Mental Health

The self-reported stress perception by the PSS demonstrated significant lower scores after the four weeks’ intervention. With regard to the BSI, the total score, the subscale somatization, and the subscale depression demonstrated significant lower values after the four weeks’ intervention. In addition, the physical score of SF-12 was significantly improved by the intervention (see Table 1).

**TABLE 1:** BSI, PSS, and SF-12 before and after four weeks intervention of HRV-BF in healthy adults.

	Mobile HRV-BF with 0.1 Hz breathing 4-weeks intervention			
BSI	Pre, M (SD)	Post, M (SD)	*t*	*p*
Total score	7.20 (6.72)	5.32 (4.75)	2.540	**≤0.05** *d = 0.51*
***Subscales***				
Somatization	2.32 (2.90)	1.40 (1.94)	3.192	**≤0.05** *d=0.64*
Depression	2.40 (2.72)	1.32 (1.73)	2.961	**≤0.05** *d=0.59*
Anxiety	2.48 (2.42)	2.60 (2.18)	−0.270	0.79
**PSS**				
Total score	14.48 (5.23)	12.20 (5.74)	2.196	**≤0.05** *d=0.44*
**SF-12**				
Physical score	51.63 (7.85)	55.24 (3.16)	−2.172	**≤0.05** *d=−0.43*
Mental score	50.32 (8.99)	50.86 (8.33)	−0.249	0.81

*BSI, Brief Symptom Inventory; PSS, Perceived Stress Sclae; SF, Short Form Health Survey.*

### Sleep Quality

As shown in Table 2, there were significant improvements in the PSQI total score as well as in the sub-scores sleep quality, sleep latency, and daytime dysfunction after the four weeks’ intervention. Concerning the actigraphy data, the use of a dependent t-test demonstrated no significant differences after the four weeks’ intervention (see Table 2).

**TABLE 2:** Objective and Subjective Sleep Quality before and after four weeks intervention of HRV-BF in healthy adults.

	Mobile HRV-BF with 0.1 Hz breathing 4-weeks intervention		*t*	*p*
Actigraphy data	Pre, M (SD)	Post, M (SD)		
Sleep, minutes	462 (63)	461 (50)	0.042	0.97
Awake, minutes	17 (13)	18 (15)	−0.071	0.94
Sleep, minutes	444 (62)	444 (45)	0.059	0.95
%-Sleep	96 (3)	96 (3)	−0.234	0.82
Sleep Onset Latency, minutes	3.54 (2.75)	3.19 (2.64)	0.693	0.50
Number of awakenings	9.83 (6.18)	11.04 (8.19)	−0.944	0.35
Wake After Sleep Onset (WASO), minutes	12.73 (10.79)	12.19 (9.79)	0.359	0.72
**PSQI**				
Total score	5.96 (2.13)	4.40 (2.15)	4.927	**≤0.001** *d=0.99*
***Subscores***				
Sleep quality	1.16 (0.62)	0.80 (0.50)	2.823	**≤0.01** *d=0.57*
Sleep latency	1.08 (0.57)	0.80 (0.41)	3.055	**≤0.01** *d=0.61*
Sleep duration	0.20 (0.41)	0.08 (0.28)	1.365	0.19
Habitual sleep efficiency	0.40 (0.58)	0.32 (0.63)	0.700	0.49
Sleep disturbances	0.96 (0.35)	1.00 (0.00)	−0.569	0.57
Use of sleeping medication	0.16 (0.47)	0.04 (0.20)	1.809	0.08
Daytime dysfunction	2.00 (1.08)	1.36 (1.00)	3.720	**≤0.01** *d=0.74*

### Autonomic Function

Concerning the 5-min sitting position, a significant increase in ln + 1 TP and ln + 1 LF could be observed after the four weeks’ intervention. With regard to the 5-minute supine position, there was a significant increase in ln + 1 SDNN, ln + 1 TP, and ln + 1 LF after the four weeks’ intervention (see Table 3).

**TABLE 3:** Heart rate and HRV-parameters before and after four weeks intervention of HRV-BF in healthy adults.

Time	Parameter	Mobile HRV-BF with 0.1 Hz breathing 4-weeks intervention				
		Pre, M (SD)	Post, M (SD)	Δ_Post–Pre_, M (SD)	*t*	*p*
5-minutes sitting position	Heart rate (b/min)	72.01 (13.67)	72.92 (10.95)	0.91 (7.33)	−0.636	0.53
	rMSSD (ms)	45.81 (19.69)	48.59 (24.60)	2.77 (18.30)		
	ln + 1 rMSSD^†^	3.76 (0.45)	3.81 (0.42)	0.06 (0.39)	−0.729	0.47
	SDNN (ms)	61.50 (19.35)	70.71 (27.24)	9.21 (22.43)		
	ln + 1 SDNN^†^	4.09 (0.32)	4.21 (0.36)	0.12 (0.33)	−1.896	0.07
	TP (ms^2^)	1363.16 (836.60)	2162.47 (2063.04)	799.31 (1673.52)		
	ln + 1 TP^†^	7.03 (0.63)	7.35 (0.81)	0.32 (0.70)	−2.339	**≤0.05** *d=−0.46*
	VLF (ms^2^)	409.32 (304.19)	425.81 (343.97)	16.49 (392.96)		
	ln + 1 VLF^†^	5.76 (0.74)	5.80 (0.72)	0.04 (0.83)	−0.233	0.82
	LF (ms^2^)	541.70 (482.47)	1296.91 (1665.97)	755.21 (1606.89)		
	ln + 1 LF^†^	5.99 (0.78)	6.62 (1.09)	0.63 (0.89)	−3.597	**≤0.001** *d=−0.71*
	HF (ms^2^)	412.14 (387.53)	439.75 (731.52)	27.61 (612.91)		
	ln + 1 HF^†^	5.63 (0.92)	5.49 (1.00)	−0.13 (1.06)	0.645	0.53
5-minutes supine position	Heart Rate (b/min)	63.29 (11.46)	65.07 (9.90)	1.78 (7.96)	−1.142	0.26
	rMSSD (ms)	67.93 (47.86)	71.41 (39.30)	3.47 (31.76)		
	ln + 1 rMSSD^†^	4.05 (0.59)	4.17 (0.48)	0.12 (0.44)	−1.356	0.19
	SDNN (ms)	74.39 (40.33)	85.02 (30.16)	10.63 (29.54)		
	ln + 1 SDNN^†^	4.22 (0.45)	4.40 (0.35)	0.18 (0.36)	−2.564	**≤0.05** *d=−0.50*
	TP (ms^2^)	2208.46 (2846.68)	2664.18 (1832.26)	455.72 (2287.67)		
	ln + 1 TP^†^	7.28 (0.85)	7.67 (0.69)	0.39 (0.74)	−2.702	**≤0.05** *d=−0.53*
	VLF (ms^2^)	480.09 (341.14)	423.25 (251.59)	−56.84 (297.35)		
	ln + 1 VLF^†^	5.92 (0.76)	5.89 (0.59)	−0.03 (0.66)	0.224	0.83
	LF (ms^2^)	992.09 (1952.18)	1480.89 (1395.26)	488.80 (1855.09)		
	ln + 1 LF^†^	6.14 (1.09)	6.83 (1.04)	0.70 (1.19)	−2.986	**≤0.01** *d=−0.59*
	HF (ms^2^)	736.28 (996.26)	760.04 (1324.38)	23.76 (902.65)		
	ln + 1 HF^†^	5.99 (1.08)	6.10 (0.91)	0.12 (0.83)	−0.704	0.49

*Δ, delta values; HF, power in high frequency range 0.15–0.4 Hz; LF, power in low-frequency range 0.04–0.15 Hz; M, mean; rMSSD, root-mean-square successive differences; SD, standard deviation; SDNN, standard deviation of all NN intervals; TP, total power; VLF, power in very low-frequency range 0.003–0.04 Hz.*

*† values were transformed by logarithm naturalis +1.*

## Discussion

This is the first study to investigate the effect of HRV-BF training on both subjective and objective sleep quality as well as on HRV in healthy adults. HRV-BF training demonstrated an improvement in the subjective sleep quality but not in the objective sleep quality (hypothesis 1) as well as an increase in the time and frequency domain parameters SDNN, Total Power and LF during two resting conditions (hypothesis 2). In addition, the mobile HRV-BF training improved mental health including somatic and depressive symptoms as well as perceived stress.

Several studies have demonstrated that HRV-BF training improves different aspects of sleep quality in individuals with depression (Lin et al., 2019), cancer (Burch et al., 2020), overactive bladder syndrome (Park et al., 2019) or caregivers with sleep disturbances (Hasuo et al., 2020). In studies with healthy adults, HRV-BF training showed an increase in the HF component during two-night recordings (Sakakibara et al., 2013). In addition, no effect of paced breathing for 20 min before going to sleep on polysomnography data during the evaluation of two consecutive nights could be observed in self-reported good sleepers (Tsai et al., 2015). After a four-week-intervention of HRV-BF training the present study showed an improvement of the subjective sleep quality with an increase in the vagal activity of healthy adults. The lack of effect on the objective sleep quality can be explained by the relatively good pre-interventional sleep patterns of our study cohort who had an average of approximately 7 1/2 h total sleep time and an average sleep efficiency of 96%. It must be taken in consideration that the effect of HRV-BF on sleep parameters was investigated in good sleepers.

We propose two potential mechanisms by which HRV-BF may improve the sleep quality of healthy adults. First, HRV-BF with 0.1 Hz breathing causes a 0° phase relation between heart oscillations and breathing, resulting in maximal RSA amplitude (Lehrer and Gevirtz, 2014). The RSA is regulated by vagal efferent pathways from the nucleus ambiguous (Yasuma and Hayano, 2004) and is described as an intrinsic resting function which increases during non-REM sleep (Bonnet and Arand, 1997). In contrast, acute psychological stress may reduce parasympathetic and increase sympathetic ANS activity, potentially resulting in a disturbed RSA function during sleep (Hall et al., 2004). There is evidence that parasympathetic dominance is important for restorative sleep (Hayano and Yasuma, 2003). Therefore, it can be argued that HRV-BF training may enhance the synchronization and efficiency of the RSA during non-REM sleep as well as it may protect the RSA function by means of vagal activation during sleep after stressful days.

Second, HRV-BF with 0.1 Hz breathing increases the baroreflex activity through the 180° phase relationship between heart rate and blood pressure (Lehrer and Gevirtz, 2014). Lehrer et al. (2003) demonstrated possible neuroplasticity of the baroreflex measured by increased resting baroreflex gain after three months of HRV-BF training. During sleep, the baroreflex is also involved in the cardiovascular autonomic modulation with, for example, an increase in the arterial blood pressure during the change from non-REM to REM sleep. Sleep deprivation and poor sleep quality are associated with impaired baroreflex sensitivity and impaired sympatho-vagal balance (Bertisch et al., 2016; Oliveira-Silva et al., 2020). Interestingly, baroreflex activity also buffers sympathetic activation during sleep, especially during cyclically alternating patterns (Iellamo et al., 2004). Due to the association between baroreflex and sleep quality, it is conceivable that HRV-BF training may improve sleep quality through an increase in baroreflex activity. In addition, the baroreflex is connected to the amygdala by the nucleus tractus solitarius (Volz et al., 1990; Polson et al., 2007), a major hub for emotional gating. Also, several studies have demonstrated positive effects on depression and anxiety through HRV-BF (Siepmann et al., 2008; Herhaus et al., 2021). Vagal afferent pathways also project to brain areas which mediate mood and emotional regulation (Grundy, 2002). Our data provide further support for a possible effect by means of lower somatic and depressive symptoms as well as lower perceived stress after four weeks of HRV-BF intervention.

The strengths of this pilot study are the rather reliable assessment of the mobile HRV-BF training compliance based on physiological (instead of self-reported) data, the high ECG-data quality with a low rate of losses (<0.06%), and the evaluation of sleep quality with objective and subjective measures. However, several limitations of the current study should be pointed out. Concerning the short-term resting condition and home HRV-BF training, breathing was neither recorded nor checked. Another limitation is the absence of sleep stage classification as an intrinsic drawback of the actigraphy method, which only analyzes sleep-wake cycles. However, it must be considered that actigraphy is a suitable research instrument recommended by the American Academy of Sleep Medicine (AASM) for measuring and evaluating sleep behavior in the home setting. With regard to the shift from sympathetic to parasympathetic dominance during the non-rapid eye movement (non-REM) sleep with a temporary increase in sympathetic system activity during REM sleep cycles, HRV-recordings during sleep before and after the intervention are missing.

In conclusion, the present data suggest that mobile HRV-BF intervention with 0.1 Hz breathing increases the subjective sleep quality and may enhance vagal activity in healthy individuals. In view of the association of impaired sympatho-vagal imbalance in sleep disorders or sleep disturbances, HRV-BF training emerges as a potentially useful treatment option for normalizing the HRV reduction to improve sleep quality and symptom severity in various sleep disorders.

## Data Availability Statement

The raw data supporting the conclusions of this article will be made available by the authors, without undue reservation.

## Ethics Statement

The studies involving human participants were reviewed and approved by Local Ethics Committee of the Landesärztekammer Rheinland-Pfalz. The participants provided their written informed consent to participate in this study.

## Author Contributions

BH: conceptualization, methodology, formal analysis, data curation, writing– original draft, and visualization. AK: data curation, and formal analysis. HG: writing – review and editing, and supervision. KP: conceptualization, methodology, writing – review and editing, and supervision. All authors revised the manuscript and approved its final version.

## Conflict of Interest

The authors declare that the research was conducted in the absence of any commercial or financial relationships that could be construed as a potential conflict of interest.

## Publisher’s Note

All claims expressed in this article are solely those of the authors and do not necessarily represent those of their affiliated organizations, or those of the publisher, the editors and the reviewers. Any product that may be evaluated in this article, or claim that may be made by its manufacturer, is not guaranteed or endorsed by the publisher.
